# Supplementary material for: Characterization and Pathogenicity of Mannheimia glucosida Isolated from Sheep
Source: Microorganisms. 2025 Nov 25;13(12):2676. doi: 10.3390/microorganisms13122676 (PMC12735675; doi:10.3390/microorganisms13122676)
Supplement: Supplementary file 1 [file microorganisms-13-02676-s001.zip › Table S1.pdf]

Table S1 All primers used in this study.

Primer	Sequence
Identification for *M. glucosida*	
LKT-F	GCAGGAGGTGATTATTAAAGTGG
LKT-R	CAGCAGTTATTGTCATACCTGAAC
LKT2-F	CTCTCTTTAGAAAAGCTGGAAAAC
LKT2-R	TTTTGCCAAGTGGTGTATTGC
HP-F	CGAGCAAGCACAATTACATTATGG
HP-R	CACCGTCAAATTCCTGTGGATAAC
bglA-F	ATGAAATTCCGTTGGGCTTAG
bglA-R	CTTTATCGTAAGCACCCAGTCC
Detection for virulence gene	
gcp-F	CGCCCCTTTTGGTTTTCTAA
gcp-R	GTAAATGCCCTTCCATATGG
gs60-F	GCACATTATATTCTATTGAG
gs60-R	AGGCATACTCTAACTTTTGC
tbpA-F	TGCCCAATATACCAAAGGCG
tbpA-R	CGATAAGTTCGGCTAAGTGT
tbpB-F	CTACTTGCTGCTTGTTCCTC
tbpB-R	AGAACCGCTTACTGTACGTC
lktC-F	GGAAACATTACTTGGCTATGG
lktC-R	TGTTGCCAGCTCTTCTTGATA
nmaA-F	CTGTAGAAGCCGGAACAGTA
nmaA-R	CATCGCCATAAGGGTTGTGA
adh-F	CTGCAAGTAAGGCAACATTG
adh-R	GAATCCGCACCAATAGCAAT
plpD-F	GACGGTTAGGGCGTAAACAA
plpD-R	TACCGTGAAAATGGCGTACA
Amplification for *lktA*	

LktA1-F	TCAAGAAGAGCTGGCAAC
LktA1-R	GCCAATGCKGTATTRATTGC
LktA2-F	GCGATTAGCCCATTAKCATT
LktA2-R	TACCGCCATCGATAAARTCA
LktA3-F	TCBGCDAAMGGBATTATTGA
LktA3-R	GTGAGGGCDACTAAACC
Amplification for *16S rRNA*, *infB*, *sodA*	
27 F	AGAGTTTGATCCTGGCTCAG
1492R	GGTTACCTTGTTACGACTT
infB-F	ACCTCGTTACTTGACTACATTCG
infB-R	ATACCGCATTCCATACCGTTAC
sodA-F	CTTATGATGCGTTAGAGCCACAT
sodA-R	GGTAGTTTAAGTAGTAAGCGTGTTC
