# Supplementary material for: Characterization and Pathogenicity of Mannheimia glucosida Isolated from Sheep
Source: Microorganisms. 2025 Nov 25;13(12):2676. doi: 10.3390/microorganisms13122676 (PMC12735675; doi:10.3390/microorganisms13122676)
Supplement: Supplementary file 1 [file microorganisms-13-02676-s001.zip › Table S2.pdf]

Table S2 Biochemical characterization of isolated strains.

Test	Isolates				
	D251	G2	G3	G4	G5
APPA	−	−	−	−	−
ADO	+	+	+	+	+
PyrA	−	−	−	−	−
IARL	+	−	−	−	+
dCEL	+	+	+	+	+
BGAL	−	−	−	−	−
H_2S	−	−	−	−	−
BNAG	−	−	−	−	−
AGLTp	−	−	−	−	−
dGLU	+	+	+	+	+
GGT	−	−	−	−	−
OFF	−	−	−	−	−
BGLU	+	+	+	+	+
dMAL	+	+	+	+	−
dMAN	+	+	+	+	+
dMNE	−	−	−	+	−
BXYL	−	−	−	−	+
BALap	−	−	−	−	−
ProA	−	−	−	−	−
LIP	−	−	−	−	−
PLE	+	+	+	+	−
TyrA	−	−	−	−	−
URE	−	−	−	−	−
dSOR	−	+	+	−	+
SAC	+	+	+	+	+
dTAG	+	−	+	+	−
dTRE	+	+	+	+	−
CIT	−	−	−	−	−
MNT	−	−	−	−	−
5KG	−	−	−	−	−
ILATk	−	−	−	−	−
AGLU	−	−	−	−	−
SUCT	−	−	−	−	−
NAGA	−	−	−	−	−
AGAL	−	−	−	−	−
PHOS	+	−	−	+	+
GIyA	−	−	−	−	−
ODC	−	−	−	−	−
LDC	−	−	−	−	−
IHISa	−	−	−	−	−
CMT	−	−	−	−	−

BGUR	−	−	−	−	−
O129R	−	−	−	−	−
GGAA	−	−	−	−	−
IMLTa	−	−	−	−	−
ELLM	−	−	−	−	−
ILATa	−	−	−	−	−

APPA: Ala-Phe-PrO-ARYLAMIDASE; ADO: ADONITOL; PyrA: L-PyrrolydonyI-ARYLAMIDASE; IARL: L-ARABITOL; dCEL: D-CELLOBIOSE; BGAL: BETA-GALACTOSIDASE; H2S: H2S PRODUCTION; BNAG: BETA-N-ACETYL-GLUCOSAMINIDASE; AGLTp: Glutamyl Arylamidase pNA; dGLU: D-GLUCOSE; GGT: GAMMA-GLUTAMYL-TRANSFERASE; OFF: FERMENTATION; BGLU: BETA-GLUCOSIDASE; dMAL: D-MALTOSE; dMAN: D-MANNITOL; dMNE: D-MANNOSE; BXYL: BETA-XYLOSIDASE; BALap: BETA-Alanine arylamidase pNA; ProA: L-Proline ARYLAMIDASE; LIP: LIPASE; PLE: PALATINOSE; TyrA: Tyrosine ARYLAMIDASE; URE: UREASE; dSOR: D-SORBITOL; SAC: SACCHAROSE; dTAG: D-TAGATOSE; dTRE: D-TREHALOSE; CIT: CITRATE; MNT: MALONATE; 5KG: 5-KETO-D-GLUCONATE; ILATk: L-LACTATE alkalinisation; AGLU: ALPHA-GLUCOSIDASE; SUCT: SUCCINATE alkalinisation; NAGA: Beta-N-ACETYL-GALACTOSAMINIDASE; AGAL: ALPHA-GALACTOSIDASE; PHOS: PHOSPHATASE; GIyA: Glycine ARYLAMIDASE; ODC: ORNITHINE DECARBOXYLASE; LDC: LYSINE DECARBOXYLASE; IHISa: L-HISTIDINE assimilation; CMT: COURMARATE; BGUR: BETA-GLUCORONIDASE; O129R: O/129 RESISTANCE; GGAA: Glu-Gly-Arg-ARYLAMIDASE; IMLTa: L-MALATE assimilation; ELLM: ELLMAN; ILATa: L-LACTATE assimilation; +: Positive; −: Negative.
