# Supplementary material for: Characterization and Pathogenicity of Mannheimia glucosida Isolated from Sheep
Source: Microorganisms. 2025 Nov 25;13(12):2676. doi: 10.3390/microorganisms13122676 (PMC12735675; doi:10.3390/microorganisms13122676)
Supplement: Supplementary file 1 [file microorganisms-13-02676-s001.zip › Table S3.pdf]

Table S3 Information on the lktA genotype in *M. glucosida*.

Strain	Genotype	Host	Geographic location	Serotype	GeneBank accession number
M. glucosida					
PH344	lktA4.1	Ovine	UK	A11	AF314517
PH498	lktA4.2	Ovine	UK	A11	AF314518
PH240	lktA4.3	Ovine	UK	A11	AF314519
PH496	lktA4.4	Ovine	UK	A11	AF314520
PH574	lktA4.5	Ovine	UK	A11	AF314521
PH290	lktA4.6	Ovine	UK	A11	AF314521
M. haemolytica					
PH8	lktA1	Ovine	UK	A1	AF314506
PH56	lktA1	Ovine	UK	A8	AF314505
PH202	lktA2	Bovine	UK	A2	AF314513
PH494	lktA2	Ovine	UK	A2	AF314511
PH196	lktA3	Ovine	UK	A2	AF314512
PH588	lktA6	Ovine	UK	A13	AF314510
PH706	lktA7	Ovine	UK	A16	AF314509
PH292	lktA8	Ovine	UK	A2	AF314515
PH296	lktA8	Ovine	UK	A7	AF414141
PH66	lktA9	Ovine	UK	A14	AF314508
PH278	lktA10	Ovine	UK	A2	AF314514
