# Supplementary material for: Characterization and Pathogenicity of Mannheimia glucosida Isolated from Sheep
Source: Microorganisms. 2025 Nov 25;13(12):2676. doi: 10.3390/microorganisms13122676 (PMC12735675; doi:10.3390/microorganisms13122676)
Supplement: Supplementary file 1 [file microorganisms-13-02676-s001.zip › Table S4.pdf]

Table S4 Information on housekeeping genes in strains.

Strain	House-keeping gene	Host	Geographic location	GeneBank accession number
M. glucosida				
P731	*16S rRNA*	Ovine	Danmark	AF053888
P733	*16S rRNA*	Ovine	Danmark	AF053892
P737	*16S rRNA*	Ovine	Danmark	AF053891
P925	*16S rRNA*	Ovine	Danmark	NR-024896
UT18	*16S rRNA*	Ovine	Danmark	AF053890
BR1441	*16S rRNA*	Ovine	Australia	KT222023
CCUG38457	*16S rRNA*	Ovine	Danmark	NR-115214
9-2234-5	*16S rRNA*	Ovine	USA	KC542331
9-2234-43	*16S rRNA*	Ovine	USA	KC542332
9-2234-45	*16S rRNA*	Ovine	USA	KC542330
8-3368-9	*16S rRNA*	Ovine	USA	KC542329
P741	*16S rRNA*	Ovine	Danmark	DQ301922
H62	*16S rRNA*	Ovine	Danmark	DQ301921
P933	*16S rRNA*	Ovine	Danmark	DQ301923
CCUG38457	*infB*	Hurse	Switzerland	DQ410887
CIP106603	*sodA*	Human	France	AY702510
M. haemolytica				
NCTC 9380	*16S rRNA*	Ovine	Danmark	NR-114448
GDZJcattle2014	*16S rRNA*	cattle	China	KM576848
38599	*16S rRNA*	Bos taurus	USA	CP017521
33041	*16S rRNA*	Bos taurus	USA	CP017501
NCTC9380	*infB*	Hurse	Switzerland	AY508847
NCTC10643	*infB*	Unkonwn	UK	LR134495
22549	*infB*	Bos taurus	USA	CP017519
1609	*infB*	Bos taurus	USA	CP017520
193	*infB*	Bos taurus	USA	CP023043
CIP103426	*sodA*	Human	France	AY702512
39433	*sodA*	Bos taurus	USA	CP017484
39409	*sodA*	Bos taurus	USA	CP017485
39394	*sodA*	Bos taurus	USA	CP017486
184	*sodA*	Bos taurus	USA	CP006957
D171	*sodA*	Bos taurus	USA	CP006573
28488	*sodA*	Bos taurus	USA	CP017506
22549	*sodA*	Bos taurus	USA	CP017519
1609	*sodA*	Bos taurus	USA	CP017520
NCTC10609	*sodA*	Unkonwn	UK	LS483299
185	Whole-genome	Bos taurus	USA	CP004753
1329	Whole-genome	Bos taurus	USA	CP017549

2286	Whole-genome	Bos taurus	USA	CP006619
M. granulomatis				
ATCC 49244	*16S rRNA*	Hare	Danmark	NR-024899
ATCC 49244	*infB*	Unkonwn	USA	EF055564
CIP103433	*sodA*	Human	France	AY702511
BJ1680.3	Whole-genome	Hare	Danmark	CP016606
M. ruminalis				
HPA92	*16S rRNA*	Ovine	Danmark	NR-024898
CCUG38470	*infB*	Horse	Switzerland	DQ410889
CIP106062	*sodA*	Human	France	AY702513
M. varigena				
177	*16S rRNA*	Bovine	Danmark	NR-024897
1388	*infB*	Bos taurus	USA	CP006953
CIP106064	*sodA*	Human	France	AY702514
1312	Whole-genome	Bovine	USA	NZ-CP006944
P. multocida				
PM8-6	*16S rRNA*	Sus scrofa	China	CP040918
CCUG17977	*infB*	Horse	Switzerland	EF055566
UNMSM alpaca	*sodA*	Unkonwn	Peru	KP660556
60248	Whole-genome	Bos taurus	USA	NZ-CP015568
