# Supplementary material for: Characterization and Pathogenicity of Mannheimia glucosida Isolated from Sheep
Source: Microorganisms. 2025 Nov 25;13(12):2676. doi: 10.3390/microorganisms13122676 (PMC12735675; doi:10.3390/microorganisms13122676)
Supplement: Supplementary file 1 [file microorganisms-13-02676-s001.zip › Table S5.pdf]

Table S5 Prediction of virulence factors in *M. glucosida*.

VFclass	Virulence factors	Related genes	D251
Adherence	BslA	*bslA*	–
	HMW1 (*Haemophilus*)	*hmw1C*	+
	Hsp60 (*Legionella*)	*htpB*	+
	Listeria adhesion protein (*Listeria*)	*lap*	+
	P5 protein (*Haemophilus*)	*ompP5*	+
	Phosphoethanolamine modification (*Neisseria*)	*lptA*	+
	Polar flagella (*Aeromonas*)	*flmH*	+
	Streptococcal plasmin receptor/GAPDH (*Streptococcus*)	*plr/gapA*	+
	Type IV pili (*Haemophilus*)	*comE/pilQ*	+
		pilA	+
		pilB	+
		pilC	+
Enzyme	Immune inhibitor A metalloproteinase	Undetermined	–
		inhA	–
	Phosphatidylcholine-preferring phospholipase C (PC-PLC)	*plcA*	–
	Phosphatidylinositol-specific phospholipase C (PI-PLC)	*piplc*	–
	Sphingomyelinase (SMase)	*sph*	–
	Streptococcal enolase (*Streptococcus*)	*eno*	+
Immune evasion	B. cereus exo-polysaccharide (BPS)	*bpsA*	–
		bpsB	–
		bpsC	–
		bpsD	–
		bpsE	–
		bpsF	–
		bpsG	–

		bpsH	–
		bpsX	–
		hasA	–
	Hyaluronic acid (HA) capsule	*hasB*	–
		hasC	–
		capA	–
		capB	–
	Polyglutamic acid capsule	*capC*	–
		capD	–
		capE	–
	Polysaccharide capsule	Undetermined	+
	Capsule (*Acinetobacter*)		+
		ctrA	+
		ctrB	+
	Capsule (*Neisseria*)	*ctrC*	+
		ctrD	+
		lipA	+
		lipB	+
	Capsule (*Streptococcus*)	*neuB*	+
		galU	+
	Exopolysaccharide (*Haemophilus*)	*manB*	+
		mrsA/glmM	+
		pgi	+
	LPS (*Brucella*)	*acpXL*	+
		dhbA	–
		dhbB	–
Iron acquisition	Bacillibactin		
		dhbC	–
		dhbE	–

		dhbF	–
	Hal	*hal*	–
	IlsA	*ilsA*	–
	Petrobactin	*asbA*	–
		asbB	–
		asbC	–
		asbD	–
		asbE	–
		asbF	–
	AcpAB	*acpA*	–
		acpB	–
Regulation	AtxA	*atxA*	–
	PagR-XO1	*pagR-XO1*	–
	PagR-XO2	*pagR-XO2*	–
	PlcR-PapR quorum sensing	*papR*	–
		plcR	–
	Carbon storage regulator A (*Legionella*)	*csrA*	+
Secretion system	Type VII secretion system	-	–
		-	–
		essC	–
		esxB	–
		esxL	–
	T4SS effectors (*Coxiella*)		+

	T6SS-II (*Klebsiella*)		+
		cya	−
	Anthrax toxin	*lef*	−
		pagA	−
	Anthrolysin O/Cereolysin O/Hemolysin I	*alo*	−
		cesA	−
		cesB	−
		cesC	−
	Cereulide	*cesD*	−
		cesH	−
		cesP	−
Toxin		*cesT*	−
	Certhrax	*cer*	−
	Cytotoxin K (Hemolysin IV)	*cytK*	−
	Hemolysin II	*hlyII*	−
	Hemolysin III homolog	Undetermined	−
	Hemolysin III	*hlyIII*	−
		hblA	−
	Hemolytic enterotoxin HBL	*hblC*	−
		hblD	−
	Insecticidal crystalline toxins	*cry*	−
		cyt	−

		vip	−
		nheA	−
	Non-hemolytic enterotoxin (Nhe)	*nheB*	−
		nheC	−
		hlyB	+
	Alpha-hemolysin (*Escherichia*)	*hlyC*	+
		hlyD	+
Amino acid and purine metabolism	Purine synthesis (*Mycobacterium*)	*purC*	+
	Alginate regulation (*Pseudomonas*)	*algU*	+
Antiphagocytosis	Capsular polysaccharide (*Vibrio*)	*rmlB*	+
		wecC	+
	Capsule (*Klebsiella*)	*gnd*	+
			+
		gmhA/lpcA	+
		htrB	+
		kdkA	+
		kdsA	+
		kdsB	+
		kdtA	+
		kpsF	+
		lgtF	+
		lpxA	+
		lpxB	+
Endotoxin	LOS (*Haemophilus*)	*lpxC*	+
		lpxD	+
		lpxH	+
		lpxK	+
		lsgA	+
		lsgD	+
		lsgE	+
		lsgF	+
		msbA	+
		msbB	+
		opsX/rfaC	+
			+
		orfM	+

		rfaD	+
		rfaE	+
		rfaF	+
		waaQ	+
		wecA	+
	Cytochrome c maturation (ccm) locus (*Legionella*)	*ccmF*	+
	Ferric enterobactin transport protein A / ferric-repressed protein B (*Neisseria*)	*fetA/frpB*	+
	Ferrous iron transport (*Shigella*)	*sitB*	+
		sitD	+
	Haemophilus iron transport locus (*Haemophilus*)	*hitA*	+
		hitB	+
		hitC	+
		hemA	+
		hemB	+
		hemC	+
Iron uptake		*hemD*	+
		hemE	+
		hemG	+
	Heme biosynthesis (*Haemophilus*)	*hemH*	+
		hemL	+
		hemM	+
		hemN	+<br>+
		hemX	+
		hemY	+
	Iron/manganese transport (*Escherichia*)	*sitA*	+
		sitC	+
	Pyoverdine (*Pseudomonas*)	*pvdH*	+
Nutritional factor	Allantoin utilization (*Klebsiella*)		+
Nutritional virulence	Biotin metabolism (*Francisella*)	*bioB*	+
Quorum sensing	Autoinducer-2 (*Vibrio*)	*luxS*	+
Serum resistance	LPS rfb locus (*Klebsiella*)	*glf*	+
Serum resistance and immune evastion	Capsule (*Francisella*)		+
	LPS (*Francisella*)		+
Stress adaptation	Catalase (*Neisseria*)	*katA*	+
